# Comparing the influence of big data resources on medical knowledge recall for staff with and without medical collaboration platform

**DOI:** 10.1186/s12909-023-04926-6

**Published:** 2023-12-13

**Authors:** JunYi Yuan, Linhui Mi, SuFen Wang, Yuejia Cheng, Xumin Hou

**Affiliations:** 1 Shanghai Chest Hospital, Shanghai Jiao Tong University School of Medicine, 241 West Huaihai Road, Shanghai, China; 2 Glorious Sun School of Business and Management, Donghua University, 1882 West Yanan Road, Shanghai, China

**Keywords:** Big data resources, Recall of prior medical knowledge, Medical collaboration platform

## Abstract

**Background:**

This study aims to examine how big data resources affect the recall of prior medical knowledge by healthcare professionals, and how this differs in environments with and without remote consultation platforms.

**Method:**

This study investigated two distinct categories of medical institutions, namely 132 medical institutions with platforms, and 176 medical institutions without the platforms. Big data resources are categorized into two levels—medical institutional level and public level—and three types, namely data, technology, and services. The data are analyzed using SmartPLS2.

**Results:**

(1) In both scenarios, shared big data resources at the public level have a significant direct impact on the recall of prior medical knowledge. However, there is a significant difference in the direct impact of big data resources at the institutional level in both scenarios. (2) In institutions with platforms, for the three big data resources (the medical big data assets and big data deployment technical capacity at the medical institutional level, and policies of medical big data at the public level) without direct impacts, there exist three indirect pathways. (3) In institutions without platforms, for the two big data resources (the service capability and big data technical capacity at the medical institutional level) without direct impacts, there exist three indirect pathways.

**Conclusions:**

The different interactions between big data, technology, and services, as well as between different levels of big data resources, affect the way clinical doctors recall relevant medical knowledge. These interaction patterns vary between institutions with and without platforms. This study provides a reference for governments and institutions to design big data environments for improving clinical capabilities.

**Supplementary Information:**

The online version contains supplementary material available at 10.1186/s12909-023-04926-6.

## Background

The diagnosis and treatment ability of medical personnel is the foundation for ensuring the high quality of medical services. Wang et al. proposed that whether medical personnel have the ability to recall relevant medical knowledge in a timely manner during the actual complex diagnosis and treatment process it is the key to ensuring their diagnosis and treatment ability [1]. Baeninger et al. showed that the minimum knowledge of keratoconus expected by corneal experts by Swiss general ophthalmologists directly leads to the number of cases of keratoconus diagnosed by ophthalmologists. Due to the low knowledge of minimum keratoconus possessed by general ophthalmologists, relatively few cases of keratoconus were diagnosed, resulting in low nursing efficiency and delayed intervention [2].

In China, because of uneven distribution of medical resources, the government and various medical institutions have been building collaborative medical services through telemedicine. During this period, many organizations have built remote consultation platforms themselves, such as medical conjoined diabetic foot intelligent diagnosis and treatment platform [3], Xinhua Chongming Medical Association ultrasound intelligent medicine [4]. There are also third-party remote platforms for collaborative medical treatment, such as remote medical consultation platform, to explore the prevention and control role of New Coronavirus’s remote medical treatment in epidemic areas [5]. With the development of collaborative medical services, medical staff will be forced to face a more complex medical service environment [6]. However, for two scenarios: using remote consultation platform and not using remote platform, whether there are differences in the influencing factors of the recall of priori medical knowledge in the provision of medical services and have not been well explored.

At the same time, the advancement and implementation of information technology and artificial intelligence have greatly contributed to the creation of medical big data, which is owned by specific medical institutions and shared across various institutions [7–9]. With the gradual accumulation of medical big data, it has further triggered policies and regulations on how to utilize various big data technologies to bring the value of medical big data into play, and how to improve the more effective and safe use of medical big data. Big data technology, medical big data, various policies and regulations are integrated into medical big data resources from both the organizational and public levels [10, 11], affecting and changing the learning and diagnosis environment of medical personnel. Wang et al. (2022) has explored the impact of medical big data resources on clinicians to recall the prior knowledge, and shown that big data technology, big data itself and big data service at the public level and institutional level interact and influence each other to activate prior medical knowledge [1]. However, it remains unclear how medical big data resources can be effectively utilized and how they may impact clinicians in two scenarios: when using remote consultation platforms, and when not utilizing any remote platform to access prior knowledge. The goal of this study is to explore how big data resources (big data technology, medical big data itself, policies and regulations) at two levels (institutional level and public level) affect clinicians to recall the prior knowledge in two different scenarios, especially the differences of these effects (including direct effects and indirect effects).

### The recall of prior medical knowledge (RPMK)

Continuous improvement of healthcare professionals’ diagnostic and therapeutic abilities is a process that is often referred to as learning. The information processing theory posits that learning involves a series of transformations of external information into knowledge through the integration of prior knowledge stored in the learner’s memory system [12]. The learner’s ability to process new external information and retrieve relevant prior knowledge from memory greatly influences the success of this cognitive process, which results in the acquisition and development of new abilities [13, 14]. The level of prior knowledge affects problem-solving strategies, as people with higher levels of prior knowledge in network search scenarios are more likely to change navigation strategies frequently to generate more effective information retrieval strategies [15]. Therefore, the ability to recall relevant knowledge is a key condition for learning to occur [14].

When healthcare professionals diagnose and treat patients, they draw upon their prior medical knowledge. This knowledge includes understanding symptoms, conducting examinations, and knowing when to prescribe medication. This knowledge is acquired through education, training, and previous experiences. With the constantly evolving medical landscape, healthcare professionals must be able to quickly activate their relevant medical knowledge to make accurate diagnoses and treatment plans. The ability to do so is critical to providing high-quality patient care. A study by Sulaiman et al. showed that complex high-quality patient care often requires the use of interdisciplinary knowledge, and the level of preparation for interdisciplinary knowledge among medical and health science students has a positive and significant impact on the perceived nursing effect of patients [16].

According to the cognitive information processing theory, humans store past experiences and knowledge in long-term memory (LTM), which has a large storage capacity. However, recalling and using relevant information from LTM can be challenging. Kiesewetter et al. showed that medical students were found to struggle with using whole-case and series-clue formats due to cognitive overload, resulting in decreased accuracy in diagnosis [17]. To assist learners in recalling prior knowledge, various information technologies have been investigated by scholars [14, 18], including web-based computer-aided education [19] and text mining technology [20], as well as virtual reality [18]. Healthcare professionals need to retrieve and activate relevant medical knowledge from long-term memory (LTM) when recalling previous medical knowledge. Similarly, scholars have paid attention to the ability of information technology to enhance healthcare professionals’ ability to review prior knowledge. Arents et al. explored the effects of 360-degree virtual reality videos on improving medical students’ long-term memory of minor cesarean section and general obstetric knowledge [21].

### Big data resources

For the value realization of medical big data [22], big data resources are divided into medical big data itself, big data technology and big data service capabilities, which are distributed at two levels: institutional level and public level [1, 23].

#### The medical big data assets (MBDA) at the level of medical institutions

As a data element at the level of medical institutions for realizing the value of big data, medical big data assets (MBDA) refer to those owned by a specific medical institution and composed of all kinds of medical big data in the hospitals, such as electronic medical records and clinical data, diagnostic data from laboratories and radiology departments, etc. [7, 8].

Big data has been defined in various ways based on the characteristics of the data generated [24]. Laney [25] proposed the 3 V model, which included Volume, Velocity, and Variability, while Manyika et al. [26] added the Value attribute to form the 4 V model. Kuo et al. [27] proposed the 5 V model, which added diversity and authenticity to the characteristics of big data. Other scholars, such as Sivarajah et al. have paid attention to the valence and volatility of big data [28]. Yaqoob et al. defined “health big data” as a large and diverse collection of biological, clinical, environmental, and lifestyle information related to an individual’s health and health status [29]. This paper highlights that the main elements of big data assets in healthcare institutions are the completeness, reliability, integration, and visualization of medical data, which serve as indicators of the quality of the medical big data owned by the institution.

The completeness of healthcare big data (CHBD) is a multidimensional index that takes into account the variety of medical data from different sources [24, 25] and the variability of the medical data context [28, 30]. Similarly, the reliability of healthcare big data (RHBD) refers to the authenticity or veracity of the data, and the lack thereof can negatively impact the quality of the big data [31]. The integration of healthcare big data (IHBD) is measured by its relevance, which is determined by the connectivity of the data across various information systems [28, 32]. Visualization of healthcare big data (VHBD) involves interpreting and identifying the most important information for users [32, 33].

The quality of medical big data is very important [34], and will affect hospital financing/reimburse men and the reuse in epidemiological or health service research [35, 36]. It will also have an important impact on improving the quality of care provided to patients, reducing access gap, improving patients’ physical condition, and better allocating resources [37]. Wang et al. (2022) has shown that medical big data assets at the level of medical institution have a significant direct impact on the activation of prior knowledge among clinicians [1].

#### The technical capacity of big data deployment (TCBD) at the level of medical institutions

As a technological element at the level of medical institutions for realizing the value of big data, the technical capacity of big data deployment in healthcare institutions refers to the ability of healthcare institutions to utilize big data technologies to collect and apply medical big data within the organization.

In healthcare institutions, mobile technology and wireless networks play crucial roles in mobilizing big data collection and application [38, 39]. The degree of adoption of mobile applications (AMA) plays a crucial role in determining the efficiency and magnitude of big data generation by medical institutions [24, 26, 38, 40]. Additionally, the quality of wireless networks (QWN) can have a significant impact on the effectiveness of wireless network utilization, resulting in diverse experiences for clinicians [40]. Through the use of these technologies, medical institutions can integrate the subject and object of big data, thereby enabling the collection and application of big data in an effective and efficient manner.

The big data value chain typically centers around human behavior, starting with the generation of big data through various means such as smart devices or mobile devices recording human activities. At the other end of the chain, big data is leveraged to actively influence or adjust human behavior. The true value of big data lies in its correlation with specific human behaviors. Wang et al.(2022) have shown that although the deployment capability of big data technology at the medical institution level does not directly affect the recall of previous medical knowledge, the deployment environment of big data technology indirectly affects the activation of relevant medical knowledge by clinical doctors based on the quality environment of big data, the big data service environment at the medical institution level, and the big information sharing environment at the public level [1].

#### The service capability of big data (SCBD) at the level of medical institutions

As a service element at the level of medical institutions for realizing the value of big data, the big data service capability of healthcare institutions refers to their ability to effectively use big data technology to fully leverage their medical big data assets.

In order to improve medical service management and enhance healthcare capacity through the use of big data, it is crucial that medical personnel have an awareness of big data [41]. However, this requires the implementation of training mechanisms [42] and the assistance of big data experts [43]. Moreover, the analysis and unlocking of the value of big data necessitates the involvement of trained professionals such as engineers, computer scientists, and statisticians, as highlighted by Fang et al. [10]. The responsibility of realizing the full potential of big data lies with these experts. Additionally, the unique challenges presented by medical big data, including patient privacy protection [44] and the establishment of proper authorization mechanisms [44, 45], also contribute to the big data service capability of medical institutions. Therefore, the availability of big data talent (ABDT), awareness of medical big data's potential (AMBD), relevant training mechanisms (TMBD), and proper authorization mechanisms for accessing and using medical big data (AMAU) are all factors that collectively contribute to the ability of healthcare institutions to harness the power of big data and maximize its impact on healthcare outcomes. Wang et al. (2022) has shown that service capability of big data at the level of medical institution has a significant direct impact on the activation of prior knowledge among clinicians [1].

#### Shared medical big data (SMBD) at the public level

As a data element at the public level for realizing the value of big data, publicly shared medical big data refers to medical research data and diagnostic and treatment data that can be shared by various institutions. The development of big data technology and the practice of various collaborative medical services have not only promoted the generation and application of medical big data within medical institutions, but also facilitated the accumulation and application of medical big data at the public level, including publicly shared diagnostic and treatment data (SDTD) and medical research data (SMRD).

Sharing diagnostic and treatment data (SDTD) with other medical institutions is often a key factor in promoting collaborative medical services and can bring many explicit benefits, including timely and effective improvement of diagnostic accuracy, better communication and coordination between doctors and patients, reduction of duplicate treatment, and mitigation of the risk of medical errors. By accessing patients’ complete treatment records through government or third-party platforms, doctors can quickly summarize the patient’s condition, reduce the patient’s medical expenses, and avoid adverse medical events such as drug interactions and contraindications [46, 47].

Obtaining research data from others is necessary for various medical research areas, including clinical effectiveness research, new drug development, and basic medical research. As medical research continues to innovate and refine, sharing research data, methods, and results is becoming increasingly important. There are many shared and free medical research databases worldwide, such as the NIH’s ECG database, Brain-CODE, and AD big data [48], which have advanced relevant medical research fields. Integrating research data from multiple medical institutions can overcome limitations of scientific research and improve doctors’ scientific research capabilities. With the advent of precision medicine, more knowledge-sharing methods have emerged, enhancing subject diagnosis and treatment abilities. Wang et al. (2022) indicate that shared medical big data at the public level has a significant direct impact on the activation of prior knowledge among clinicians [1].

#### The policies of medical big data (PMBD) at the public level

As a service element at the public level for realizing the value of big data, public-level medical big data policies refer to public policies and regulations that promote the accumulation and application of medical big data within a region to fully realize its value. These policies will affect the realization of the value of medical big data in the diagnosis and treatment processes of various medical institutions [32]. The “Healthy China 2030 Planning Outline” issued by the State Council of China proposes to promote data tracking in the entire series of processes such as medical treatment, drug procurement, consumables access, and chronic disease tracking, and break data silos through regional medical authorities to develop medical data sharing standards and real-time data reporting by medical institutions at various stages, thus achieving the collaboration of medical services. Many regional platforms have collected massive data, which fully meet the conditions for discovering new knowledge, creating new value, and enhancing new capabilities, further supporting the development of the healthcare industry. Public policies related to medical big data, as an important component of the public service environment, are important factors in forming the value of medical big data. Wang et al. (2022) found that although the public service environment at the public level does not directly affect the recall of prior medical knowledge, it is also based on the big data quality environment, the big data service environment at the medical institution level, and big data sharing environment at the public level, indirectly affecting the activation of relevant medical knowledge by clinical doctors [1].

### Telemedicine

In China, the healthcare system has faced long-standing challenges of uneven distribution and insufficient supply of medical resources across different levels of care. As a result, primary medical institutions often struggle to meet the healthcare needs of local residents. To address this issue, the establishment of “Internet plus medical” and telemedicine systems can provide primary healthcare providers with the capacity to conduct remote pathology and telemedicine consultations for patients while also receiving support from superior hospitals. A survey in Sichuan province revealed the substantial need for telemedicine services among healthcare personnel. The most in-demand services included remote consultation (90.3%), remote pathology (68.1%), remote training (66.1%), remote imaging (58.5%), and remote ECG (55.5%). These findings underscore the importance of providing grass-roots healthcare providers with remote consultation and communication services for pathology, imaging, and ECG to improve patient outcomes [49].

In order to accelerate the development of remote healthcare and improve its quality, the United States and the European Union attach great importance to promoting the progress of remote healthcare through the issuance of policy regulations [50]. Many scholars are concerned about issues such as the quality of remote healthcare services and their influencing factors. For example, Sherwood et al. studied the safety and effectiveness of male prisoner urology remote healthcare projects through retrospective research methods. They believe that remote healthcare can solve the problems of 90% of patients and is a safe and effective medical approach [51]. Rasekaba et al. studied the impact of remote healthcare interventions on mothers and fetuses with gestational diabetes mellitus through meta-analysis methods [52].

Furthermore, telemedicine services promote collaborative medical services, which can enhance the service abilities of medical staff on the telemedicine platform. Without video conferencing, diagnostic pathways (such as visual and clinical examination) may be lost in the interaction between cardiologists and family doctors [53]. Traditional written referrals often lead to incomplete information, which can negatively impact the quality and comprehensiveness of communication [6].

When using a remote consultation platform for medical services, multiple medical staff members collaborate to provide care. Whether they are requesting or providing the service, they must ask and answer various questions during the diagnosis and treatment process with other medical staff members. This creates a distinct scenario for medical services compared to not using a remote consultation platform. However, little research has focused on whether this difference in service scenarios affects the recall of prior knowledge required to complete the medical services, and how it may impact the outcome.

### This study

The literature review indicates that big data resources will affect clinicians’ review of prior medical knowledge [1], and the use of remote medical platforms has changed clinicians’ diagnosis and treatment service processes [6, 29]. However, there has been no detailed exploration of the differences in the influence of big data resources on RPMK in two scenarios, i.e., when using a self-built or third-party remote consultation platform versus not using a remote platform. This study seeks to explore and comprehend how different types of big data resources, at both institutional and public levels, affect healthcare professionals’ medical knowledge review in two different scenarios, especially to identify any variations in the impact mechanisms (including direct effects and indirect effects) of these resources between the two scenarios.

**Fig. 1:**
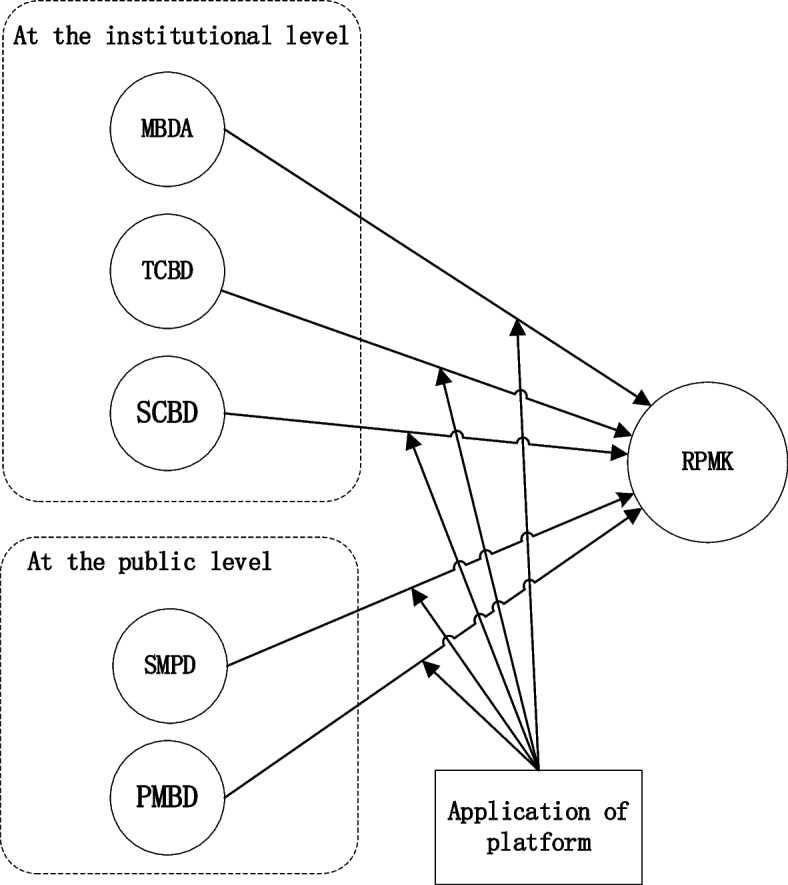
Conceptual model of the impact of big data resources on RPMK

Based on the literature discussed above, and is depicted in the conceptual model in Fig. 1, we hypothesize that the impact of medical big data assets (MBDA), technical capacity of big data deployment (TCBD) and the service capability of big data (SCBD) at the level of medical institutions, shared medical big data (SMBD) and the policies related to medical big data (PMBD) at the public level on the medical knowledge review of healthcare professionals is influenced by whether the institution has used remote medical platforms.

## Methods

### Participants and procedures

This study was conducted on 308 hospitals in China. Among them, 132 institutions used either self-built or third-party remote consultation platforms, while 176 did not use any self-built or third-party remote consultation platforms (seen Table 1). The response rate was 308/360 (86%). Because the main body of providing healthcare services are public hospitals in China, public hospitals and a limited number of private hospitals have been primarily targeted for the study.

**Table 1:** Type and level of hospital

	With platform	Without platform
Type of hospital		
Public hospitals	126 (95.5%)	157 (89.2%)
Private hospitals	6 (4.5%)	19 (10.8%)
total	132 (100%)	176 (100%)
Hospital level		
Tertiary general hospitals	39 (29.5%)	38 (21.6%)
Tertiary specialty hospitals	13 (9.8%)	26 (14.8%)
Second-class general hospitals	75 (56.8%)	76 (43.2%)
Second-class specialty hospitals	4 (3.0%)	17 (9.7%)
Community hospitals	1 (0.8%)	18 (10.2%)
Others	0 (0.0%)	1 (0.6%)
total	132 (100%)	176 (100%)

In the survey process, two groups of people are involved in each institution. The first group serves as the study contacts. Each institution has a contact person whose tasks include: (1) confirming the interest of the target medical institution in participating in the survey, (2) selecting survey respondents from that institution, and (3) distributing and collecting questionnaires. The second group comprises the survey questionnaire respondents. There are two respondents from each medical institution, one is a medical staff and the other is an IT executive staff. During the design process of the items (see Measurement scales for details), it was found that medical staffs in medical institutions are not familiar with the relevant content of the measurement items of the policies of medical big data (PMBD) at the public level, making it difficult to answer. However, IT executive staffs understand and can answer the relevant items. Therefore, the survey questionnaire for each institution is divided into two parts, each completed by a medical staff or an IT executive staff within the institution.

The medical staff in each target hospital responded to survey questions related to MBDA, TCBD, SCBD, and SMBD, while the IT executive staff provided information regarding the PMBD. Additionally, information technology personnel answered whether the organization has utilized self-built or third-party remote consultation platforms, as the development and use of such platforms require the support of IT staff. So, respondents included 308 IT staffs and 308 medical staffs. The sample profile is shown in Table 2.

**Table 2:** Demographic information of respondents

	IT personnel		Medical personnel	
	With platform	Without platform	With platform	Without platform
Sex				
Male	83 (62.9%)	139 (79.0%)	77 (58.3%)	139 (79.0%)
Female	49 (37.1%)	37 (21.0%)	55 (41.7%)	37 (21.0%)
total	132 (100%)	176 (100%)	132 (100%)	176 (100%)
Age				
20–30	35 (26.5%)	62 (35.2%)	15 (11.4%)	29 (16.5%)
31–40	79 (59.8%)	90 (51.1%)	75 (56.8%)	104 (59.1%)
41–50	18 (13.6%)	22 (12.5%)	36 (27.3%)	39 (22.2%)
51–60	0 (0.0%)	2 (1.1%)	6 (4.5%)	4 (2.3%)
total	132 (100%)	176 (100%)	132 (100%)	176 (100%)
Education degree				
High school	8 (6.1%)	3 (1.7%)	5 (3.8%)	1 (0.6%)
Bachelor	114 (86.4%)	154 (87.5%)	77 (58.3%)	107 (60.8%)
Master	10 (7.6%)	19 (10.8%)	48 (36.4%)	64 (36.4%)
Doctorate	0 (0.0%)	0 (0.0%)	2 (1.5%)	4 (2.3%)
total	132 (100%)	176 (100%)	132 (100%)	176 (100%)

The specific data collection plan is designed as follows: (1) Query the relevant contacts of the target medical institution through WeChat and ask if they are willing to participate in the investigation. (2) The questionnaire distribution is mainly distributed and collected in the form of paper questionnaires. However, for medical institutions with long distances, with the consent of the contact of the institution, the questionnaire is sent and collected through WeChat. However, it is not directly sent to two respondents in the institution, but is forwarded and forwarded through the contact of the institution to maximize the protection of the respondents’ personal privacy. The questionnaire was administered between August 1, 2017, and October 31, 2017.

The study protocol was reviewed and approved by the Ethical Review Committee at the Shanghai Chest Hospital. Before conducting the research, all participants were informed in writing of the purpose and procedure of this study. Participants can voluntarily choose not to participate in this study. The confidentiality and anonymity of all participants’ collected information were ensured.

### Measurement scales

Orlikowski and Iacono [54] proposed five perspectives for IT articles: tool view, proxy view, ensemble view, computational view, and nominal view. Proxy view is the most commonly used perspective in IT articles research. The tool view of the big data resources reflects people’s positioning of big data resources and what role they hope to play in collaborative medical service. The proxy perspective of the big data resources mainly reflects people’s perception of its existence. The ensemble view is to emphasize the dynamic formation process of the big data resources from the perspective of design science. The computational view includes technology as an algorithm and technology as a model. In the computing view, we pay more attention to the model and algorithm itself. The nominal view often happens when we forget about technology and present big data technology and big data as a pure background.

The application process of big data resources is the interaction process of various views of big data resources, that is, the computational view (i.e. algorithms and models of big data resources) forms the tool view of big data resources (i.e. the formation of various decision-maker roles) through the ensemble view (i.e. dynamic development process), which is abbreviated as the nominal view of big data resources at the macro level. These four views of big data resources can be materialized into various attributes that decision-makers can perceive, forming a proxy perspective for big data resources. Decision makers either perceive its existence by accepting certain characteristics or performance, or realize its existence by sensing its diffusion or spread in various medical institutions, industries, and economies, or perceive its existence by discovering its financial investment [54]. Therefore, the proxy perspective is that various stakeholders in the social environment where big data resources are located perceive their existence through various means, and reflects how the service based on big data resources is accepted and realized.

Based on the proxy view proposed by Orlikowski and Iacono [54], this study explores the medical big data resources and designs the items. In order to ensure content validity, the items for big data assets, big data deployment technical capability and big data service capability at the level of medical institutions, and public-level sharing of medical big data were expanded and modified from previous research [55–58]. Additionally, the items for big data policy and regulations at the public level (PMBD) were self-developed.

The item design stage involved field interviews and group discussions, with a working group consisting of five members. This group included a director of the information center, an information center staff member, a director of the medical department, a director of the outpatient office, and a clinician. The group discussed and revised the items, paying particular attention to those with vague descriptions that might lead to ambiguity in understanding. A total of 62 declarative sentence items were formed for the pilot test, all measured on a 7-point Likert scale. These items are available in the Supplementary material.

Four aspects of medical big data assets (MBDA) at the institutional level were evaluated, namely completeness of healthcare big data (CHBD), reliability of healthcare big data (RHBD), integration of healthcare big data (IHBD), and visualization of healthcare big data (VHBD). The diversity and scope of medical information owned by medical institutions were assessed through three items to measure CHBD (with platform: Cronbach’s α = 0.916; M = 5.71, SD = 1.06; without platform: Cronbach’s α = 0.925; M = 5.18, SD = 1.38). The authenticity, correctness, consistency, and absence of contradiction in the medical information were assessed through four items to measure RHBD (with platform: Cronbach’s α = 0.949; M = 5.42, SD = 1.07; without platform: Cronbach’s α = 0.969; M = 5.06, SD = 1.34). The degree to which healthcare data from various information systems are connected was assessed through four items to measure IHBD (with platform: Cronbach’s α = 0.972; M = 5.36, SD = 1.30; without platform: Cronbach’s α = 0.963; M = 4.97, SD = 1.42). Lastly, the visualization of healthcare data was assessed based on the degree to which the content and format of information are easily understandable was measured through four items to evaluate VHBD (with platform: Cronbach’s α = 0.842; M = 5.47, SD = 0.99; without platform: Cronbach’s α = 0.922; M = 5.04, SD = 1.25) (seen in Table 3).

**Table 3:** Mean, SD, Cronbach’s α of constructs on two datasets

Constructs	Dimensions & Items	with platform			without platform		
		Mean	SD	Cronbach’s α	Mean	SD	Cronbach’s α
MBDA	CHBD (3 items)	5.707	1.062	0.916	5.184	1.377	0.925
	RHBD (4 items)	5.422	1.074	0.949	5.064	1.337	0.969
	IHBD (4 items)	5.364	1.298	0.972	4.966	1.422	0.963
	VHBD (4 items)	5.470	0.992	0.842	5.044	1.249	0.922
TCBD	ABDT (4 items)	4.539	1.826	0.977	3.438	1.808	0.977
	AMBD (3 items)	4.737	1.644	0.920	3.913	1.690	0.928
SCBD	AMAU (4 items)	5.527	1.079	0.914	5.057	1.331	0.932
	TMBD (4 items)	5.278	1.366	0.947	4.587	1.461	0.928
	AMA (5 items)	5.498	1.248	0.939	5.122	1.217	0.951
	QWN (4 items)	5.218	1.300	0.914	5.105	1.128	0.912
SMBD	SDTD (7 items)	4.517	1.645	0.988	3.736	1.629	0.991
	SNRD (3 items)	4.747	1.635	0.975	4.047	1.506	0.976
PMBD	3 items	5.485	1.159	0.930	4.735	1.353	0.967
RPMK	5 items	4.994	1.300	0.960	4.468	1.392	0.971

*MBDA* Medical Big Data Assets at the level of medical institutions, *TCBD* Technical Capacity of Big Data deployment at the level of medical institutions, *SCBD* Service Capability of Big Data at the level of medical institutions, *SMBD* Shared Medical Big Data at the public level, *PMBD* Policies of Medical Big Data at the public level, *RPMK* Recall of Prior Medical Knowledge

The technical capacity of big data deployment (TCBD) at the level of medical institutions was evaluated with respect to two key aspects, namely, the degree of adoption of mobile applications (AMA) and t the quality of wireless networks (QWN). The scope, function, and ease of use of mobile applications were assessed through five items to measure the coverage of mobile applications (AMA) (with platform: Cronbach’s α = 0.977; M = 4.54, SD = 1.83; without platform: Cronbach’s α = 0.977; M = 3.44, SD = 1.81). The coverage, stability of operation, speed, and security of wireless networks were assessed through four items to measure QWN (with platform: Cronbach’s α = 0.920; M = 4.74, SD = 1.64; without platform: Cronbach’s α = 0.928; M = 3.91, SD = 1.69) (seen in Table 3).

The study evaluated the medical big data service capabilities of medical institutions (SCBD) based on four aspects: availability of big data talent (ABDT), awareness of medical big data’s potential (AMBD), authorization mechanisms for accessing and using medical big data (AMAU), and personnel training mechanism (TMBD). The sufficiency and ability of IT professionals owned by medical institutions were assessed through four items to measure ABDT (with platform: Cronbach’s α = 0.914; M = 5.53, SD = 1.08; without platform: Cronbach’s α = 0.932; M = 5.06, SD = 1.33). AMBD was measured by three items, including the initiative, frequency, and coverage of healthcare big data usage (with platform: Cronbach’s α = 0.947; M = 5.28, SD = 1.37; without platform: Cronbach’s α = 0.928; M = 4.59, SD = 1.46). AMAU was assessed using four items that explored the existence, convenience, rapidness, and effects of information authorization procedures (with platform: Cronbach’s α = 0.939; M = 5.50, SD = 1.25; without platform: Cronbach's α = 0.951; M = 5.12, SD = 1.22). Lastly, the degree to which medical institutions provided education and training on health information systems was measured through four items to evaluate TMBD (with platform: Cronbach’s α = 0.914; M = 5.22, SD = 1.30; without platform: Cronbach’s α = 0.912; M = 5.11, SD = 1.13) (seen in Table 3).

The study evaluated the public sharing of medical big data (SMBD) in two aspects: the sharing of diagnosis and treatment data (SDTS) and the sharing of medical research data (SMRD). To assess the sharing of diagnosis and treatment data (SDTS), participants were asked to respond to seven items that measured the extent to which they could obtain diagnosis and treatment data from other medical institutions through government public platforms or third-party platforms (with platform: Cronbach’s α = 0.988; M = 4.52, SD = 1.64; without platform: Cronbach’s α = 0.991; M = 3.74, SD = 1.63). The sharing of medical research data (SMRD) was evaluated through three items that asked participants about the degree to which they could access research data from other medical institutions through third-party databases such as CNKI and PubMed (with platform: Cronbach’s α = 0.975; M = 4.75, SD = 1.64; without platform: Cronbach’s α = 0.976; M = 4.05, SD = 1.51) (seen in Table 3).

The assessment of policies and regulations related to big data (PMBD) was conducted using three items that evaluated the extent of rationality, existence, and functional completeness of relevant policies, laws, and regulations pertaining to regional medical service platforms (with platform: Cronbach’s α = 0.93; M = 5.48, SD = 1.16; without platform: Cronbach’s α = 0.967; M = 4.73, SD = 1.35) (seen in Table 3).

The study measured the recall of prior knowledge using 5 items to assess the degree to which the participants’ knowledge of drug indications, drug contraindications, pharmacokinetics, and diagnostics is activated. The internal consistency of the measurement was assessed using Cronbach’s alpha, which was found to be 0.96 with the platform and 0.971 without the platform. The mean score for recall of prior knowledge was 4.99 with the platform and 4.47 without the platform, with a standard deviation of 1.30 and 1.39, respectively (seen in Table 3). Using Kruskal Wallis test analysis, it was found that there was no significant difference in the perception of the recall of prior knowledge among medical staffs based on gender, age, level and type of hospital they were in. However, there was a significant difference in the perception of the recall of prior knowledge among medical staffs based on differences in their educational background (chi square: 8.521; *P*: 0.014). There is a significant difference in the perception of the recall of prior knowledge among medical institutions whether they have used remote medical platforms (chi square: 11.045; *P*: 0.001).

### Data analysis and procedure

**Fig. 2:**
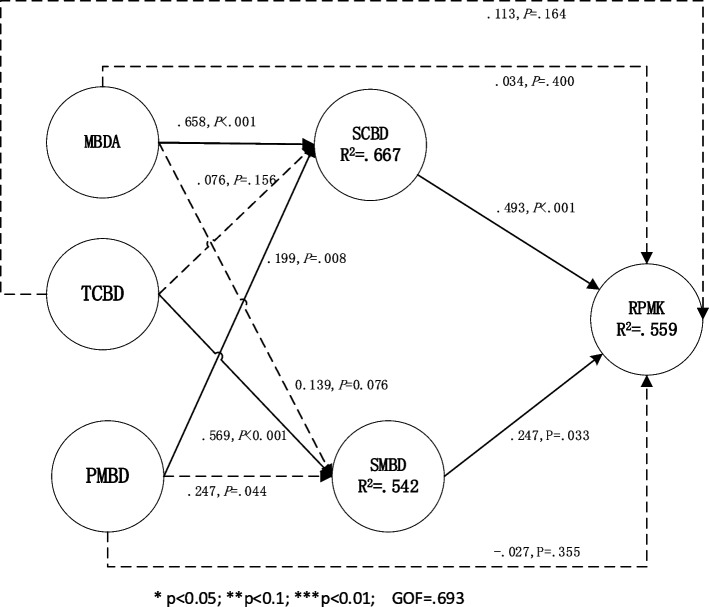
The structural model 2 on the data set with platform: including direct and indirect effects

**Fig. 3:**
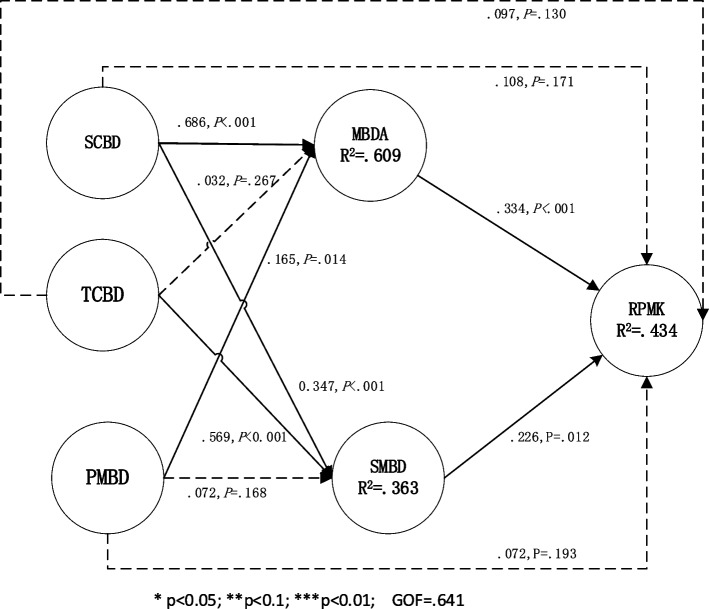
The structural model 3 on the data set without platform: including direct and indirect effect

The data analysis tool used was the SmartPLS version 2.0 software package. Data analysis mainly consists of three steps:

- Step 1: Construct model 1, which is the direct impact model of five major data resources on RPMK (refer to Fig. 1). Evaluate the measurement model characteristics of all five data resources on two datasets, including reliability and validity.
- Step 2: Using model 1 as the basis, analyze the significance of the direct impact of the five big data resources on RPMK. Additionally, examine the differences in these impacts between the two datasets.
- Step 3: Two-tier structural models 2 (see Fig. 2) and 3 (see Fig. 3) are built for data sets with and without platforms. These two models add the relationship path between big data resources that have no significant impact on the base of model 1 and those that have a direct significant impact on big data resources, so as to further analyze the intermediary effect of big data resources that have no significant impact on RPMK.

## Results

### Measurement model

Build Model 1 according to the direct impact path between the variables in Fig. 1, and run the model on two data sets respectively. Tables 4, 5 and 6 show the characteristics of the measurement model on the two datasets.

**Table 4:** Reliability and convergence validity test on two datasets

Constructs	Dimensions & Items	Load Value		CR		AVE	
		with platform	without platform	with platform	without platform	with platform	without platform
MBDA	CHBD (3 items)	0.859	0.879	0.914	0.927	0.728	0.761
	RHBD (4 items)	0.881	0.860				
	IHBD (4 items)	0.784	0.849				
	VHBD (4 items)	0.884	0.901				
SCBD	ABDT (4 items)	0.873	0.817	0.898	0.863	0.689	0.613
	AMBD (3 items)	0.828	0.774				
	AMAU (4 items)	0.815	0.795				
	TMBD (4 items)	0.802	0.742				
TCBD	AMA (5 items)	0.924	0.931	0.934	0.926	0.875	0.863
	QWN (4 items)	0.947	0.926				
SMBD	SDTD (7 items)	0.919	0.900	0.922	0.907	0.856	0.794
	SNRD (3 items)	0.931	0.921				
PMBD	3 items	0.916 ~ 0.966	0.963 ~ 0.975	0.956	0.979	0.878	0.897
RPMK	5 items	0.917 ~ 0.949	0.852 ~ 0.905	0.970	0.978	0.864	0.897

*MBDA* Medical Big Data Assets at the level of medical institutions, *TCBD* Technical Capacity of Big Data deployment at the level of medical institutions, *SCBD* Service Capability of Big Data at the level of medical institutions, *SMBD* Shared Medical Big Data at the public level, *PMBD* Policies of Medical Big Data at the public level, *RPMK* the Recall of Prior Medical Knowledge

**Table 5:** Discriminant validity test on the dataset with platform (Fornell-Larcker criterion)

	MBDA	SCBD	TCBD	SMBD	PMBD	RPMK
MBDA	0.853					
SCBD	0.790	0.830				
TCBD	0.498	0.499	0.936			
SMBD	0.492	0.543	0.708	0.925		
PMBD	0.476	0.548	0.478	0.484	0.937	
RPMK	0.588	0.695	0.537	0.598	0.433	0.930

**Table 6:** Discriminant validity test on the dataset without platform (Fornell-Larcker criterion)

	MBDA	SCBD	TCBD	SMBD	PMBD	RPMK
MBDA	0.872					
SCBD	0.763	0.782				
TCBD	0.333	0.350	0.929			
SMBD	0.449	0.496	0.489	0.911		
PMBD	0.450	0.398	0.368	0.336	0.969	
RPMK	0.585	0.540	0.385	0.503	0.381	0.947

*MBDA* Medical Big Data Assets at the level of medical institutions, *TCBD* Technical Capacity of Big Data deployment at the level of medical institutions, *SCBD* Service Capability of Big Data at the level of medical institutions, *SMBD* Shared Medical Big Data at the public level, *PMBD* Policies of Medical Big Data at the public level, *RPMK* Recall of Prior Medical Knowledge

Table 4 presents the factor loading values of all items, which are higher than 0.78 on the dataset with platform and higher than 0.74 on the dataset without platform, and are all significant. The composite reliability (CR) value is approximately 0.9 on the dataset with platform and higher than 0.86 on the dataset without platform, which exceeds the normal value of 0.7 [59, 60]. These values meet the minimum requirement for indicator reliability and internal consistency reliability on both datasets. Additionally, the average variance extracted (AVE) for all constructs is ≥ 0.689 on the dataset with platform and ≥ 0.613 on the dataset without platform, which is above the normal value of 0.5. Therefore, the model demonstrates good convergent validity on both datasets [61].

The results from Tables 5 and 6 show that the square root of the Average Variance Extracted (AVE) values for each construct are higher than the correlation coefficients between constructs. This satisfies the Fornell-Larcker criterion [61] for good discriminant validity. The results from Table 7 present HTMT values. In both datasets, the HTMT values between all pairwise constructs are less than 0.85. Hence, the measurement models for both datasets are considered to have good discriminant validity.

**Table 7:** Heterotrait-monotrait ratio (HTMT)—List

	With platform	Without platform
PMBD <—> MBDA	0.482	0.465
RPMK <—> MBDA	0.600	0.590
RPMK <—> PMBD	0.454	0.393
SCBD <—> MBDA	0.818	0.801
SCBD <—> PMBD	0.573	0.412
SCBD <—> RPMK	0.726	0.566
SMBD <—> MBDA	0.475	0.459
SMBD <—> PMBD	0.459	0.347
SMBD <—> RPMK	0.568	0.494
SMBD <—> SCBD	0.499	0.485
TCBD <—> MBDA	0.503	0.333
TCBD <—> PMBD	0.490	0.383
TCBD <—> RPMK	0.542	0.398
TCBD <—> SCBD	0.498	0.364
TCBD <—> SMBD	0.750	0.495

### Differences in direct impact paths under different data sets

Table 8 displays the path coefficient and significance of the direct impact that five big data resources have on RPMK in model 1 for both data sets. It also showcases the differences in these direct impacts between the two data sets.

**Table 8:** Differences in direct impact paths under different data sets

Path	With platform			Without platform			Difference	
	β	SE	*P*-value	β	SE	*P*-value	Z	significance
**MBDA—> RPMK**	**0.026**	**0.132**	**0.408**	**0.331**	**0.099**	**< 0.001**	**-7.708**	**yes**
**SCBD—> RPMK**	**0.491**	**0.122**	**< 0.001**	**0.113**	**0.112**	**0.159**	**9.548**	**yes**
TCBD—> RPMK	0.113	0.115	0.153	0.100	0.083	0.124	0.334	no
PMBD—> RPMK	-0.026	0.070	0.340	0.071	0.088	0.196	-3.032	yes
**SMBD- > RPMK**	**0.262**	**0.113**	**0.014**	**0.229**	**0.097**	**0.011**	**0.898**	**no**

R^2^ = 0.559 with platform; R^2^ = 0.434 with platform

*MBDA* Medical Big Data Assets at the level of medical institutions, *TCBD* Technical Capacity of Big Data deployment at the level of medical institutions, *SCBD* Service Capability of Big Data at the level of medical institutions, *SMBD* Shared Medical Big Data at the public level, *PMBD* Policies of Medical Big Data at the public level, *RPMK* Recall of Prior Medical Knowledge

In the data set with platform, the service capability of big data (SCBD) at the level of medical institutions and shared medical big data (SMBD) at the public level significantly and directly impact RPMK at the levels of 0.001 and 0.05, respectively. However, in the data set without platform, the medical big data assets (MBDA) at the level of medical institutions and big data sharing environment (SMBD) at the public level significantly and directly impact RPMK at the levels of 0.001 and 0.05, respectively. Nevertheless, there is no substantial distinction in the direct impact of SMBD between the two data sets.

### Influence path on the dataset with platform

Model 2 was developed based on the findings of model 1, which was analyzed using the dataset with platform. The analysis results of model 2 on the platform-inclusive dataset are shown in Fig. 2. The relationships between variables, as indicated by standardized regression weights and level of significance, are displayed. The coefficient of determination R2 was used to assess the extent to which latent dependent variables accounted for the total variance. A cut-off of 0.190 indicates weak explanatory power, 0.333 moderate explanatory power, and 0.670 substantial explanatory power. Specifically, the analysis revealed that RPMK and SMBD were moderately explained, with 55.9% and 54.2% of their variance accounted for, respectively. SCBD had a higher level of explanatory power, with 66.7% of its variance accounted for.

To evaluate the overall adequacy of the model, we employ the Goodness-of-Fit (GoF) criterion. In the present study, the calculated GoF for the model 2 is 0.693, which is considered to be high [62].

From Fig. 2 we can see: since MBDA, TCBD and PMBD have no direct impact on RPMK on the dataset with platform, the indirect effect of these three variables on RPMK is further analyzed. The results of the mediation test are presented in Table 9. To assess the magnitude of the indirect effects (Helm et al., 2010), the VAF (variance accounted for) value was calculated, which represented the ration of the indirect effect to the total effect.

**Table 9:**
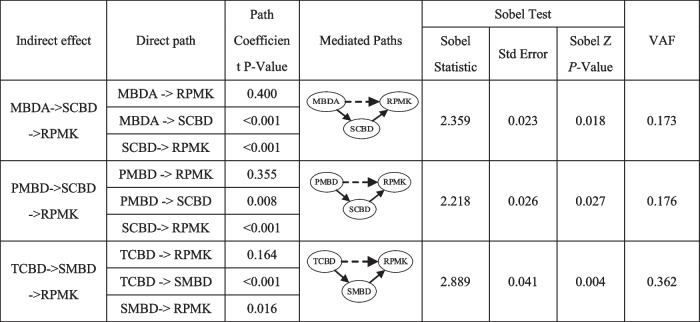
Mediating effect test on the data set with platform

*MBDA* Medical Big Data Assets at the level of medical institutions, *TCBD* Technical Capacity of Big Data deployment at the level of medical institutions, *SCBD* Service Capability of Big Data at the level of medical institutions, *SMBD* Shared Medical Big Data at the public level, *PMBD* Policies of Medical Big Data at the public level, *RPMK* Recall of Prior Medical Knowledge

From Tables 8 and 9 we can see:

- The indirect impact of MBDA. Although MBDA have no direct impact on RPMK, there is one completely mediated path on the dataset with platform: MBDA- > SCBD- > RPMK (*p* < 0.05). SCBD play the mediating role in the effect of MBDA on RPMK (VAF is 0.173). The total effect of MBDA on RPMK was 0.394, and it was significant in *P* < 0.001. However, 17.3% of the total effects are indirect effects based on SCBD. It indicates that for medical institutions to utilize self-built or third-party remote consultation platforms, they require robust internal medical data resources to enhance the quality of their medical services. This includes having a strong pool of information technology experts, proficiency in the use of big data, a robust authorization mechanism, and a comprehensive training system [17]. Ultimately, this will lead to an improved ability of medical staff to recall relevant medical knowledge in clinical practice.
- The indirect impact of TCBD. Although TCBD have no direct impact on RPMK, there are one completely mediated path on the dataset with platform: TCBD- > SMBD- > RPMK (*p* < 0.01). In the mediated path, SMBD play the mediating role in the effect of TCBD on RPMK (VAF is 0.362). The total effect of TCBD on RPMK was 0.291, and it was significant in *P* < 0.01. However, 36.2% of the total effects are indirect effects based on SMBD. It indicates that the availability of wireless networks and mobile applications in medical institutions facilitates the sharing of medical big data, as per research by Akoka et al. [38] and Gil et al. [39]. This promotes the sharing and utilization of diagnostic and treatment data, as well as public research data when using self-built or third-party remote consultation platforms. Ultimately, this leads to an improved ability of medical staff to recall relevant medical knowledge during clinical practice.
- The indirect impact of PMBD. Although PMBD have no direct impact on RPMK, there are three completely mediated path on the dataset with platform: PMBD—> SCBD- > RPMK (*p* < 0.05). In the mediated path, SCBD play mediating roles in the effect of PMBD on RPMK (VAF is 0.176). It indicates that public-level policies and regulations concerning big data (PRPMBD) have enhanced the development of a big data service environment in medical institutions [63]. This has created a platform for medical professionals to utilize big data, making it easier for them to access relevant medical knowledge during their clinical practice.

### Influence path on the dataset without platform

Model 3 was constructed based on the findings of model 1, which was analyzed using the dataset without platform. The analysis results of model 3 on the platform-excluded dataset are displayed in Fig. 3, revealing the relationships between variables in terms of standardized regression weights and level of significance. The coefficient of determination R^2^ was utilized to measure the degree to which latent dependent variables explained the overall variance. The cut-off values are as follows: 0.190 for weak explanatory power, 0.333 for moderate explanatory power, and 0.670 for substantial explanatory power. Specifically, the analysis revealed that RPMK, SMBD, and MBDA had moderate explanatory power, with 43.4%, 36.3%, and 60.9% of their variance accounted for, respectively. Lastly, the calculated GoF for the model 3 is 0.641, which is considered high [62].

From Fig. 3 we can see: since SCBD, TCBD and PMBD have no direct impact on RPMK, the indirect effect of these three variables on RPMK is further analyzed. The results of the mediation test are presented in Table 10.

**Table 10:**
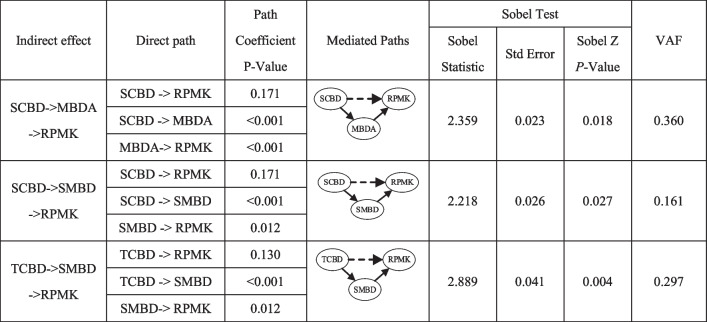
Mediating effect test on the data set without platform

*MBDA* Medical Big Data Assets at the level of medical institutions, *TCBD* Technical Capacity of Big Data deployment at the level of medical institutions, *SCBD* Service Capability of Big Data at the level of medical institutions, *SMBD* Shared Medical Big Data at the public level, *PMBD* Policies of Medical Big Data at the public level, *RPMK* Recall of Prior Medical Knowledge

From Table 8 and 10, we can see:

- The indirect impact of SCBD on the dataset without platform. Although SCBD have no direct impact on RPMK, there are two completely mediated path on the dataset without platform: SCBD- > MBDA- > RPMK (*p* < 0.05) and SCBD- > SMBD- > RPMK (*p* < 0.05). In these paths, MBDA and SMBD play mediating roles in the effect of SCBD on RPMK (VAF is 0.711). The total effect of SCBD on RPMK was 0.32, and it was significant in *P* < 0.01. However, 71% of the total effects are indirect effects based on MBDA and SMBD. In other words, if a medical institution is not using a self-built or third-party remote consultation platform, they can still enhance the quality of their medical big data assets by improving their internal big data service environment. This can be achieved by strengthening their information technology talent pool, cultivating awareness among medical staff on the use of big data, establishing a more effective authorization mechanism, and implementing a comprehensive training system for medical data. Improving the quality of medical big data assets within the institution, including data integrity, credibility, integration, and understandability [28], can ultimately help medical staff better recall relevant medical knowledge and apply it in clinical practice.
- The indirect impact of TCBD on the dataset without platform. Although TCBD have no direct impact on RPMK, there are one completely mediated path: TCBD- > SMBD- > RPMK (*p* < 0.01). In the path, SMBD play mediating role in the effect of TCBD on RPMK (VAF is 0.297). The total effect of TCBD on RPMK was 0.185, and it was significant in *P* < 0.05. However, 29.7% of the total effects are indirect effects based on SMBD. It indicates that wireless network accessibility and mobile application coverage in medical institutions promote the sharing of medical big data [38, 39], ultimately helping medical staff better recall relevant medical knowledge during clinical practice.
- On the dataset without platform, PMBD has neither direct nor indirect impact on RPMK.

## Discussion

### Main findings

*The impact of the service capability of big data (SCBD) at the level of medical institutions.* When medical institutions utilize either self-built or third-party remote medical consultation platforms, SCBD can directly and significantly influence clinicians’ recall of prior medical knowledge (RPMK) and mediate the impact of medical big data assets (MBDA) within the institution and policies of medical big data (PMBD) at the public level on RPMK. In the absence of a platform, SCBD cannot directly and significantly impact RPMK, but it can indirectly affect RPMK through the influence of MBDA and PMBD, with a cumulative VAF of 0.520.

*The impact of the medical big data assets (MBDA) at the level of medical institutions.* In the context of medical institutions using self-built or third-party remote medical consultation platforms, the impact of medical big data assets (MBDA) on clinicians’ recall of prior medical knowledge (RPMK) is not straightforward. However, it can have an indirect impact through the service capability of big data (SCBD) at the institutional level, which can affect RPMK. On the other hand, in the absence of such platforms, MBDA can directly and significantly impact RPMK and also mediate the impact of SCBD on RPMK.

*The impact of the technical capacity of big data deployment (TCBD) at the level of medical institutions.* TCBD does not have a direct impact on clinicians’ recall of prior medical knowledge (RPMK), regardless of whether remote medical consultation platforms are used or not. However, there is an indirect impact on RPMK, and this impact is generated through medical big data assets (MBDA) in both scenarios. It is worth noting that the indirect impact on RPMK is significantly stronger in the scenario where remote medical consultation platforms are utilized. In contrast, in the absence of such platforms, the indirect impact on RPMK is relatively weaker.

*The impact of sharing big data (SMBD) at the public level.* Sharing big data publicly has a direct influence on RPMK, regardless of whether healthcare facilities utilize remote medical consultation platforms or not. However, this impact does not differ significantly between the two scenarios. Additionally, SMBD acts as an intermediary factor in the impact of other big data resources on RPMK. When a platform is present, it primarily serves as an intermediary factor between TCBD and RPMK. In the absence of a platform, it not only acts as an intermediary factor between TCBD and RPMK but also between SCBD and RPMK.

*The impact of the policies of medical big data (PMBD) at the public level.* The policies of medical big data (PMBD) do not directly affect the RPMK (Remote Patient Monitoring Kit) regardless of whether healthcare institutions use remote medical consultation platforms or not. However, in scenarios where platforms are used, there is an indirect impact based on the SCBD (Secondary Clinical Big Data) but no such impact in scenarios where platforms are not used. To assess the indirect impact of PMBD, new mediating factors need to be identified and studied.

### Academic implications

This study contributes several important findings to the current understanding of the subject matter.

Firstly, the study reveals that the direct impact of five big data resources on clinicians’ recall of prior medical knowledge (RPMK) is widespread. The study also shows that shared medical big data (SMBD) at the public level has a significant impact on RPMK, regardless of whether medical institutions have remote medical collaboration platforms or not. The study highlights the importance of establishing a public medical research database and promoting the sharing of diagnosis and treatment data and research data, as this can help clinicians extract relevant diagnosis and treatment knowledge and improve their ability to provide accurate and effective treatment. These findings are consistent with prior research that emphasizes the significance of medical big data shared at the public level [64]. According to the study of Daei et al. [64], clinicians are likely to face various challenges while caring for patients. However, discovering high-quality evidence in a timely and convenient manner can offer a good opportunity to enhance patient care. Their study finds that clinicians commonly seek clinical information by consulting their peers, and by simultaneously accessing journal articles, Internet websites, textbooks, as well as EDLINE / PubMed. The research findings are consistent with those of Wang et al. [1], and further confirm that shared medical big data resources at the public level have a direct and significant impact on clinicians’ recall of prior medical knowledge. At the same time, it further reveals that there is no significant difference in this impact between the two scenarios.

The research highlights the essentiality of building the technical capacity for deploying big data (TCBD) at the level of medical institutions. Despite not directly impact on clinicians’ recall of prior medical knowledge (RPMK) in either of the two scenarios, TCBD’s indirect influence on clinicians’ recall of prior medical knowledge could stem from the popularity of mobile applications and wireless technology in various medical settings, which act as a backdrop for medical service providers and facilitate the sharing of public-level medical big data. Thus, although TCBD’s impact may not be immediately tangible, its significance cannot be undermined. This conclusion is consistent with the research findings of Wang et al. [1], and further reveals that the indirect impact of TCBD on clinicians’ recall of prior medical knowledge based on SMBD is significant in two scenarios.

Secondly, our investigation has brought to light the variations in the direct impact that big data resources have on clinicians’ ability to recollect prior medical knowledge (RPMK). It is imperative for medical institutions that utilize remote medical collaboration platforms to prioritize the establishment of service capability of big data (SCBD). On the other hand, medical institutions that do not utilize such platforms should prioritize the development of their medical big data assets (MBDA). This study shares the findings of Wang et al. [1], indicating that both SCBD and MBDA at institutional level have a direct and significant impact on clinicians’ recall of prior medical knowledge. However, due to Wang et al. [1] not considering the application of two types of scenarios, this study further analyzed the situation in both scenarios and found significant differences in the direct impact of SCBD and MBDA at institutional level on doctor knowledge review between the two scenarios.

In healthcare organizations that utilize remote medical collaboration platforms, the medical big data assets (MBDA) at the institutional level do not have a direct impact on clinicians’ recall of prior medical knowledge (RPMK). However, the service capability of big data (SCBD) at the institutional level has a significant positive direct effect on clinicians’ RPMK. In other words, as medical institutions engage in more collaborative medical and external healthcare organization services, more technological and service support is needed. Medical service personnel with a better understanding of big data are more willing to use medical big data to solve problems during the diagnosis and treatment process. Sufficient and excellent big data professionals in medical institutions can provide better technical support and related training for clinical doctors. Comprehensive authorization and effective training methods give clinical doctors a clear concept of the data collection scope, making it easier for them to activate relevant knowledge during the diagnosis and treatment process and ultimately improve their medical service capabilities. This finding is consistent with previous research that highlights the importance of internal and external big data service environments [65, 66]. Jimenez et al. [65] suggested that to fully utilize medical data in primary care systems, there must be human resources with digital technology capabilities and understanding. Their research also indicates that the adoption of digital tools and technologies is slow, partly due to the low digital health literacy of primary healthcare workers. Butler Henderson et al. [67] believe that cultivating high-quality specific digital health capabilities requires a good training system. The difference between this study and these previous studies is that this study focuses more on the impact of the service capability of big data (SCBD) at the institutional level on clinicians’ recall of prior knowledge to reflect its importance in healthcare organizations that utilize remote medical collaboration platforms.

However, for medical institutions that do not utilize collaborative healthcare platforms, the service capability of big data (SCBD) at the institutional level does not directly impact clinicians’ recall of prior medical knowledge (RPMK). Nonetheless, the medical big data assets (MBDA) at the institutional level significantly and positively influence clinicians’ RPMK. In other words, when medical institutions do not have direct or third-party collaboration platforms, healthcare professionals need to understand the quality of medical big data assets at the institutional level. The quality of medical big data assets at the institutional level (e.g., accuracy, completeness, integration, and comprehensibility) will greatly impact the activation of relevant knowledge during the diagnostic and therapeutic process. When medical big data quality is high, meaning that the information contained within is relatively complete, accurate, and easy to comprehend, the relevance of the diagnostic and therapeutic process is enhanced, and clinicians are better equipped to activate previously learned drug, diagnostic, and laboratory knowledge to provide better care for patients. This finding is consistent with previous research showing the importance of medical data quality at the institutional level [34]. Samadbeik et al. [34] emphasized the importance of medical data quality, which will affect hospitals’ financing/reimbursement and reuse in epidemiological or health service research [35, 36]. It will also improve the quality of care provided to patients, reduce disparities in access, improve patient outcomes, and better allocate resources [37]. However, this study further reveals the importance of the medical big data assets (MBDA) at the institutional level in medical institutions that do not use collaborative healthcare platforms.

Thirdly, the interplay of three key components involved in the realization of big data value, namely big data itself, technology, and services, impacts clinicians’ recall of relevant medical knowledge (RPMK). This study uncovers a broader range of diversified patterns of combinations among big data, big data technology, and big data service that affect RPMK, compared to prior investigations [1, 14, 20, 22]. Furthermore, the study investigates the differences in the interaction pathways of the three elements of big data value realization in two types of medical institutions: those utilizing remote medical collaboration platforms and those that do not.

The first type of interaction pathway pertains to situations where the elements and relationships are identical, but the strength of the relationships differs. Regardless of whether remote medical collaboration platforms are employed, the interaction pathway of “technology- > data- > RPMK” exists, albeit with varying degrees of intensity. The second type of interaction pathway is characterized by similar elements, but differing relationships. In healthcare institutions that use remote medical collaboration platforms, the interaction pathway is “data- > service- > RPMK”, while in those that do not, the pathway is “service- > data- > RPMK”. In institutions that employ remote medical collaboration platforms, medical big data primarily refers to the data within the institution, whereas in those that do not, it encompasses both institutional and public-level data. Therefore, in the presence of a platform, the interaction pathway between data and services is singular, whereas in the absence of a platform, two separate pathways exist. Additionally, there exists an interaction pathway of “service- > service- > RPMK” that is exclusive to institutions that use remote medical collaboration platforms. Although the policies of medical big data (PMBD) at the public level do not have a direct impact on the recall of medical knowledge in institutions that do not use these platforms, there exists an intermediary pathway (“external service- > internal service- > RPMK”) that influences the recall of clinical medical knowledge in institutions that employ these platforms. Specifically, in institutions that use remote medical collaboration platforms, PMBD indirectly affects the recall of clinical medical knowledge by influencing the service capability of big data (SCBD) at the institutional level.

Fourthly, the study reveals the diverse combination patterns of different levels of big data resources that impact clinicians’ recall of relevant medical knowledge (RPMK) and the differences in these interaction pathways between medical institutions that use remote medical collaboration platforms and those that do not, compared to prior studies [14, 68]. The interaction of different levels of big data resources involves three ways: “Institution—> Institution—> RPMK”, “Public—> Institution—> RPMK”, and “Institution—> Public—> RPMK”. However, in the two types of medical institutions, the specific performance of these three interaction modes differs. This study further confirms the conclusion of Wang et al. [1] that the interaction of different levels of big data resources affects doctors’ review of knowledge. However, this study further reveals the forms and differences of this interaction mode of medical big data resources at different levels in different scenarios, making the results of this study more targeted and instructive for different medical institutions.

The interaction mode “Institution—> Institution—> RPMK” can be found in both medical institutions using and not using remote medical collaboration platforms, but the form is the same, while the content differs. In medical institutions using remote medical collaboration platforms, the data elements of the value of big data at the institutional level (MBDA: The medical big data assets at the level of medical institutions) indirectly affect clinicians’ RPMK through the service elements at the institutional level (SCBD: the service capability of big data at the institutional level). In contrast, in medical institutions without platforms, the service elements at the institutional level (SCBD) indirectly affects clinicians’ RPMK by influencing the data elements at the institutional level (MBDA).

Only in medical institutions using remote medical collaboration platforms did the study observe the intermediate path of “Public—> Institution—> RPMK”, specifically manifested as the service element at the public level (PMBD: the policies of medical big data at the public level) indirectly affecting RPMK by influencing the service element at the institutional level (SCBD: the service capability of big data at the institutional level). However, this interaction did not significantly occur in the absence of platforms.

The interaction between “Institution- > Public- > RPMK” is significantly evident in two different scenarios, where the intermediary factor is the data element at the public level (SMBD: shared medical big data at the public level). However, the number and strength of intermediary paths differ in these two scenarios. In the platform-based scenario, there is only one intermediary path of “Institution- > Public- > RPMK,” whereas there are two intermediary paths in the non-platform-based scenario. The technology element at the institutional level (TCBD: the technical capacity of big data deployment at the level of medical institutions) indirectly influences RPMK by affecting the data element at the public level (SMBD). This interaction is found in both types of institutions, but the intermediary effect is significantly stronger in the platform-based scenario than in the non-platform-based scenario.

Moreover, in the medical institutions that do not use remote collaborative platforms, the service element at the institutional level (SCBD: the service capability of big data at the level of medical institutions) indirectly affects RPMK by influencing the data element at the public level (SMBD) via the intermediary path of “Institution- > Public- > RPMK”. However, this intermediary path is not found in the medical institutions that use remote collaborative platforms.

### Managerial implications

This study offers several practical applications for clinicians, medical institutions, and relevant government departments or companies. Firstly, clinicians can improve their ability to diagnose and treat patients by utilizing various big data resources at the institutional and public level. This can be achieved by activating prior knowledge and increasing their service ability.

Secondly, this study offers a framework for medical institutions to effectively utilize their internal medical big data assets and service capabilities of big data. The study also proposes leveraging big data sharing at the public level to facilitate clinicians in easily recalling previous medical knowledge, thereby enhancing their diagnostic and treatment capabilities and improving patients’ satisfaction with medical services. To achieve this, medical institutions can increase the collection and utilization of medical big data, improve the quality of medical big data by enhancing wireless coverage and diversifying mobile software, provide various training programs to medical staff to increase their awareness of big data, and enable them to access relevant medical knowledge quickly whenever they encounter any problems. This includes drug usage, diagnosis and treatment processes, and inspection methods.

Thirdly, this study serves as a basis for developing appropriate policies and funding directions for government departments. For example, policies could be implemented to design collaborative medical networks that specify sharing of diagnosis and treatment data, and invest in establishing public research databases to share research results. Additionally, the establishment of a public big data sharing environment can improve the ability of medical staff to recall relevant medical knowledge in clinical practice.

### Limitations and future research

Firstly, the main focus of this study is to examine how big data resources can activate prior knowledge for learning in both institutional and public settings. While this is important, it is also necessary for clinicians to enhance their diagnostic and treatment skills through learning about other conditions, such as how to effectively organize their acquired medical knowledge and how to reduce cognitive load to address limited attentional resources. To fully leverage big data resources and enhance the capabilities of medical personnel, future research should investigate their impact on other learning conditions as well.

Secondly, the research mainly compares and analyzes the impact of big data resources on medical staff’s ability to recall relevant medical knowledge during the diagnosis and treatment process in two different scenarios—with and without the use of a remote medical collaboration platform. However, medical staff’s effectiveness in providing healthcare services involves several other factors, and the learning process often requires the interaction of various conditions. Therefore, future research should focus on exploring and comparing the combined effects of different big data resources on the coordination of various learning conditions in both scenarios. This will provide us with a better understanding of how to leverage big data resources to enhance medical staff's diagnosis and treatment capabilities.

Third, this study mainly utilizes proxy view of technology proposed by Orikowski & Iacono’s [54] to observe and analyze dig data resources as perception. Future research can start from computational view of technology proposed by Orikowski & Iacono’s [54] and explore how medical big data can be processed through an algorithmic process to assist medical professionals in decision-making, especially in diagnostic assistance. Future research can also start from ensemble view of technology and approach dig data resources as production network to observe the formation of big data resources from a larger system, observing that more types of groups (including providers and rule-makers) jointly promote the formation and protection of data privacy in big data environments, and the impact on the prior knowledge review of medical staff. Future research can also be based on the ensemble view of technology and approach the dig data resources as embedded system, believing that the big data environment is an evolutionary system and dynamic formation process embedded in complex and dynamic social scenarios. Based on this perspective, the known and unknown issues of the prognosis of medical data are reflected in the big data environment to consider their impact on the prior knowledge review of medical staff.

Finally, the current study utilized a convenient sampling method for pre-testing and data collection. The semantic rationality and clear and understandable expression of the items in this study are mainly based on 62 participants from medical institutions with platforms during the pre-test. Future research can further search for testers in medical institutions that do not use the platform to further test the semantic rationality of the items. Furthermore, it is important to note that the research findings may benefit from further validation through the use of larger and more diverse sample sizes. Therefore, future research could aim to replicate the current study with a larger and more representative sample to enhance the reliability and generalizability of the results.

## Conclusion

The utilization of big data resources has a distinct and common impact on medical knowledge recall (RPMK) for healthcare professionals in medical institutions using remote medical collaboration platforms versus those that do not. Beyond direct effects, three elements of big data value realization at two levels interact to impact medical knowledge recall in five intermediate pathways for healthcare professionals. These pathways include “institutional data—> institutional services—> RPMK,” “institutional services—> institutional data—> RPMK,” “institutional technology—> public data—> RPMK,” and “public services—> institutional services—> RPMK.” Nevertheless, the specific elements of these intermediate pathways exert distinct effects in the two scenarios.

### Supplementary Information

**Additional file 1.**

## Data Availability

The datasets used during this study are available from the corresponding author on reasonable request.

## Abbreviations

SE: Standard Error
MBDA: Medical Big Data Assets at the level of medical institutions
TCBD: Technical Capacity of Big Data deployment at the level of medical institutions
SCBD: Service Capability of Big Data at the level of medical institutions
SMBD: Shared Medical Big Data at the public level
PMBD: Policies of Medical Big Data at the public level
RPMK: Recall of Prior Medical Knowledge
