# Supplementary material for: Comparing the influence of big data resources on medical knowledge recall for staff with and without medical collaboration platform
Source: BMC Med Educ. 2023 Dec 13;23:956. doi: 10.1186/s12909-023-04926-6 (PMC10720120; doi:10.1186/s12909-023-04926-6)
Supplement: Supplementary file 1 — Additional file 1. [file 12909_2023_4926_MOESM1_ESM.docx]

# Supplementary Material: Questionnaire

**Completeness of healthcare data (CHBD)**

CHBD_1: In my daily work, all kinds of information systems can provide complete information (for example, I am able to retrieve the details such as name, gender, home address, etc., when I would like to inquire patient information).

CHBD_2: In my daily work, the information provided by various information systems is very comprehensive (for example, I am able to retrieve the details such as name, bed number, delivery time, etc., when I would like to inquire laboratory report).

CHBD_3: In my daily work, the information provided by various information systems is very complete (for example, I am able to retrieve the details such as name, bed number, admission time, etc., when I would like to inquire hospitalization history).

**Reliability of healthcare data (RHBD)**

RHBD_1: The data in the report involving my daily work is consistent with the actual situation.

RHBD_2: There is no obvious wrong data in the data reports involving my daily work.

RHBD_3: There is no obvious contradiction in the data among the various reports involving my daily work.

RHBD_4: There is no obvious inconsistency in the data among the various reports involving my daily work.

**Integration of healthcare data (IHBD)**

IHBD_1: In my daily work, whether in the outpatient clinic or in the inpatient workstation, I can conveniently see all the outpatient and inpatient diagnosis and treatment information of the patient.

IHBD_2: In my daily work, whether in the outpatient clinic or in the inpatient workstation, I can flexibly see all the outpatient and inpatient diagnosis and treatment information of the patient.

IHBD_3: In my daily work, whether in an outpatient clinic or an inpatient workstation, I can conveniently see the patient diagnosis and treatment information I want to see.

IHBD_4: In my daily work, whether in the outpatient clinic or in the inpatient workstation, I can flexibly see the patient diagnosis and treatment information I want to see.

**Visualization of healthcare data (VHBD)**

VHBD_1: In my daily work, the data provided by the information system is very intuitive and clear at a glance.

VHBD_2: In my daily work, the data provided by the information system is easy to understand.

VHBD_3: In my daily work, the data format presented by the information system is concise and clear.

VHBD_4: In my daily work, the data presented to me by the information system has various forms such as graphs and tables.

**Adoption of mobile applications (AMA)**

AMA_1: Mobile application software (such as mobile follow-up, mobile ward rounds or other software adopted in my institution), has covered every step of my daily work.

AMA_2: mobile application software (such as mobile follow-up, mobile ward rounds or other software adopted in my institution), has all the functions I need.

AMA_3: I can find all the information I need in mobile applications (such as mobile follow-up, mobile ward rounds or other software adopted in my institution).

AMA_4: In my daily work, the information queried through mobile applications is relatively accurate.

AMA_5: In my daily work, mobile applications (such as mobile follow-up, mobile ward rounds or other software adopted in my institution) are relatively convenient to use.

**Quality of wireless networks (**QWN**)**

QWN_1: In my daily work area, the wireless network is fully covered.

QWN_2: At present, the hospital wireless network used for medical work is relatively stable.

QWN_3: At present, the speed of the hospital wireless network used for medical work is relatively fast.

QWN_4: I use the hospital wireless network for medical work and I think it is relatively safe.

**Availability of big data talent (ABDT)**

ABDT_1: The information technology professionals in our hospital have already possessed a relatively professional level of technical ability.

ABDT_2: The staffing of information technology professionals in our hospital is relatively sufficient.

ABDT_3: The information technology professionals in our hospital can provide timely clinical services.

ABDT_4: The information technology professionals in our hospital can well complete the bridge work between clinical needs and software modification.

**Awareness of medical big data's potential (AMBD)**

AMBD_1 When encountering problems in daily work, I will think of using data technology to help solve them in the first place.

AMBD_2 In my daily work, I often use big data technology to solve related problems.

AMBD_3 Data technology has covered every aspect of my daily work.

**Authorization management mechanism for accessing and using medical big data (AMAU)**

AMAU_1: Our hospital already has related information authorization procedures (such as prescription rights application, workload statistics, etc.).

AMAU_2: The current information authorization process (such as prescription rights application, workload statistics, etc.) is relatively convenient in our hospital.

AMAU_3: The current information authorization process (such as prescription rights application, workload statistics, etc.) from application to approval is quick in our hospital.

AMAU_4: The current information authorization process (such as prescription rights application, workload statistics, etc.) can effectively control the risk of data leakage in our hospital.

**Personnel training mechanism (TMBD)**

TMBD_1: Our hospital will conduct pre-training before use of the information system.

TMBD_2: Our hospital will hold lectures on the use of information systems regularly.

TMBD_3: The training on the use of the information system in our hospital has achieved good results.

TMBD_4: The training on the use of the information system in our hospital has been quite satisfactory.

**Sharing of diagnosis and treatment data (SDTD)**

SDTD_1: Diagnosis and treatment data of other medical institutions can be easily obtained (such as data sharing through government public platforms and medical networks).

SDTD_2: The information provided by other medical institutions' diagnosis and treatment data sharing (such as through government public platforms, medical networking) is accurate.

SDTD_3: Diagnosis and treatment data sharing of other medical institutions (such as through government public platforms, medical networking) provides more comprehensive information.

SDTD_4: The diagnosis and treatment data sharing of other medical institutions (such as through government public platforms, medical networking) effectively reduces the time of the diagnosis time of diseases.

SDTD_5: The diagnosis and treatment data sharing of other medical institutions (such as through government public platforms, medical networking) avoids repeated inspections.

SDTD_6: The diagnosis and treatment data sharing of other medical institutions (such as through government public platforms, medical networking) avoids repeated medication.

SDTD_7: Diagnosis and treatment data sharing of other medical institutions (such as through government public platforms, medical networking) avoids adverse reactions between various drugs.

**Sharing of medical research data (SMRD)**

SMRD_1: Research data from other medical institutions (CNKI, PubMed, etc.) can be easily obtained.

SMRD_2: The research data sharing of other medical institutions (CNKI, PubMed, etc.) provides relatively complete scientific research information.

SMRD_3: Research data from other medical institutions (CNKI, PubMed, etc.) are all valuable.

**Policies of medical big data at the public level (PMBD)**

PMBD_1: The relevant policies, laws and regulations on the regional medical service platform (three-level referral from the Health and Family Planning Commission, medical consortium, and regional medical treatment) are reasonable.

PMBD_2: The construction of the regional medical service platform is supported by policies, laws and regulations (three-level referral from the Health and Family Planning Commission, medical consortium, and regional medical treatment).

PMBD_3: The difficulties encountered in the construction of the regional medical service platform can be solved according to policies, laws and regulations (three-level referral from the Health and Family Planning Commission, medical consortium, and regional medical treatment).

**Recall of prior medical knowledge (RPMK)**

RPMK_1: At present, the information system of our hospital has related functions, which can help me recall the relevant knowledge of drug indications I learned before.

RPMK_2: At present, the information system of our hospital has related functions, which can help me review the relevant knowledge of drug contraindications I have learned before.

RPMK_3: At present, our hospital information system has related functions, which can help me extract the previous knowledge of pharmacokinetics.

RPMK_4: At present, the information system of our hospital has related functions, which can help me recall the diagnostics and other related knowledge that I have learned.

RPMK_5: At present, the information system of our hospital has related functions, which can help me review the diagnostic knowledge related to imaging that I have learned.
